# Rapid Evidence Review of Community Engagement and Resources in the UK during the COVID-19 Pandemic: How Can Community Assets Redress Health Inequities?

**DOI:** 10.3390/ijerph19074086

**Published:** 2022-03-30

**Authors:** Rabya Mughal, Linda J. M. Thomson, Norma Daykin, Helen J. Chatterjee

**Affiliations:** 1 Department of Genetics, Evolution and Environment, Division of Biosciences, University College London, London WC1E 6AA, UK; rabya.mughal@ucl.ac.uk (R.M.); linda.thomson@ucl.ac.uk (L.J.M.T.); 2 Music Therapy Department, UWE Bristol, Glenside Campus, Bristol BS16 1DD, UK; norma.daykin@uwe.ac.uk

**Keywords:** sociocultural, environmental health, health behaviour

## Abstract

Community engagement, such as participating in arts, nature or leisurely activities, is positively associated with psychological and physiological wellbeing. Community-based engagement during the COVID-19 pandemic facilitated informal and local mutual aid between individuals. This rapid evidence review assesses the emergence of community-based arts, nature, music, theatre and other types of cultural engagement amongst UK communities in response to the COVID-19 pandemic. Here, we focus on all community engagement with a sub-focus on provisions accessed by and targeted towards vulnerable groups. Two hundred and fifty-six resources were included that had been created between February 2020 and January 2021. Resources were identified through Google Scholar, PubMed, Web of Science, MedRXic, PsycharXiv and searches for grey literature and items in the public domain. The majority reported services that had been adapted to become online, telephone-based or delivered at a distance from doorsteps. Several quality assessment frameworks were used to evaluate the quality of data. Whilst a number of peer-reviewed, grey literature and public domain articles were identified, less than half of the identified literature met quality thresholds. The pace of the response to the pandemic may have meant that robust evaluation procedures were not always in place.

## 1. Introduction

Holistic approaches to healthcare are now well evidenced, having been utilised by community referral specialists since the mid-1990s [1,2]. Community assets such as museums, libraries and third-sector organisations can promote social health and wellbeing through art, nature, music or creative activities (hereby called ‘community activities’) [3]. Participation in community activities involves aesthetic engagement, evocation of the imagination and emotion, cognitive stimulation, sensory stimulation, social interaction and physical activity—which in turn endorse positive psychological (e.g., coping and emotional strategies), physiological (e.g., lower stress hormone response), social (e.g., reduced loneliness and isolation) and behavioural outcomes (e.g., adoption of healthier behaviours and skills development) [4]. Evidence suggests that such salutogenic approaches are useful in the treatment and prevention of long-term conditions, can take pressure off of socialised healthcare systems and can be effective in increasing resilience and wellbeing in individuals and communities [1,5]. Despite evidence of the positive impact of cultural engagement on the population in general, there remains inconsistency in the evidence for community assets as reducers of health inequity in disadvantaged, marginalised or vulnerable communities [6].

Vulnerable populations entered the COVID-19 pandemic from uneven starting points [7]. Living in poverty, receiving low wages or being a member of a single-parent household indicates likelihood of the highest levels of net COVID-19-related impact, whilst those living in areas of deprivation are more likely to be exposed to the Coronavirus [8]. These disparities put vulnerable populations at risk of negative health outcomes, which are exacerbated by already existing structural and institutional disadvantages [7,9,10]. For instance, those with chronic physiological or psychological health conditions are most likely to be disproportionately and adversely affected by viral load as well as socioeconomic impact [7]. Those accessing hospital outpatient services and those living with chronic health conditions have experienced delays to care plans and elective treatments [7,11]. In addition, the pandemic has changed the landscape of mental health services in the UK, with the increasing need for the development of telehealth services in community care [12].

The COVID-19 pandemic resulted in an increase in community-led activity seeking to support vulnerable and shielding individuals [13]. Some of these were new groups set up in response to the pandemic, whilst others were adaptations from existing services [14]. Although many services have been adapted for vulnerable individuals, less is known about the efficacy of these service adaptations, their mechanisms or their impact on vulnerable populations.

The aim of this review was to draw together a broad summary of how community activities have been utilised during the COVID-19 pandemic. This review examines how local organisations have engaged with vulnerable and non-vulnerable groups using community activities during the COVID-19 pandemic, and how efficacy has been measured by various community organisations.

## 2. Materials and Methods

A rapid evidence review approach was used to scope and assess the range of resources offered. As a result, a wide range of peer-reviewed, grey literature and public domain resources were included, providing evidence of the variety of community engagement activities on offer during the COVID-19 pandemic [15]. Inclusion criteria were: resources that outlined creative and/or nature-based activities such as arts, theatre, music, sewing, social, gaming, gardening, exercise, community and other related activities (including those using museums, libraries and other cultural assets); resources that reported intervention data on the above-mentioned activities; resources that provided contextual information on the impact of the COVID-19 pandemic on vulnerable groups; and resources that provided contextual data on the psychological, physiological and socioeconomic impact of COVID-19. All resources were written in English and published between March 2020 and January 2021. Peer-reviewed literature included intervention, longitudinal, random control trial, cross-sectional, exploratory or narrative review data. Non-peer-reviewed resources included grey literature (comprising of governmental or local authority reports, third-sector reports and study protocols) and items within the public domain (comprising web pages, blogs, social media pages and information shared within and between communities). Exclusion criteria were: resources unrelated to COVID-19; resources targeted only at schools, children and adolescents; resources not applicable to the UK population.

**Figure 1:**
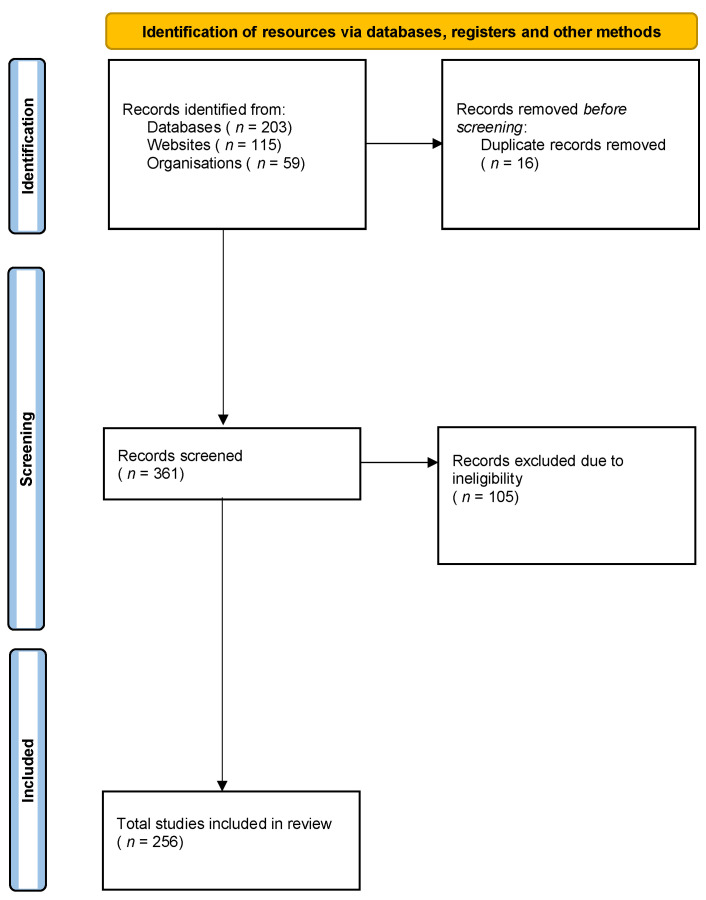
PRISMA flow diagram of searches of databases, registers and other sources.

A range of review methodologies were utilised to draw together a broad summary of the current evidence for the efficacy of community-based interventions on vulnerable populations. Here, ‘vulnerability’ is categorised by physical, psychological or socioeconomic circumstances as defined by the Office of National Statistics [16]. An overview of the number of resources identified, screened and included can be seen in Figure 1.

### 2.1. Search Protocol

Searches for peer-reviewed literature were carried out on Google Scholar, PubMed, Web of Science, MedRXiv and PsycharXiv (see Appendix A for search terms). Non-peer-reviewed grey literature and public domain resources were identified using hand searching in Google, reference lists and input from the wider project team and its affiliated organisations: the Culture Health and Wellbeing Alliance [17], Arts Council England [18], Natural England [19] and UKRI March Mental Health Research Network [20]. Government and statistical reports were identified via the UK government [21] and Office for National Statistics [22] websites.

### 2.2. Quality Assessment

Several quality assessment frameworks were utilised for the purposes of evaluating the quality of resources collected for this review. Examples of activities, case studies that formed part of grey literature resources and public domain resources were quality assessed using a tool devised from the Public Health England (PHE) Arts for Health and Wellbeing evaluation framework [23]. Resources that provided more than 10 out of 15 relevant PHE Arts for Health and Wellbeing evaluation criteria met this review’s quality assurance threshold. Grey literature reports were evaluated using AACODS (authority, accuracy, coverage, objectivity, date and significance) critical appraisal [24]. Grey literature that scored in the top third (>22 out of 34) of the AACODS criteria met this review’s quality assurance threshold. Peer-reviewed literature was evaluated using AMSTAR [25] for reviews and Cochrane Quality Appraisal [26] for intervention, cross-sectional or regression data. The literature was strategically sampled and checked by all four authors.

### 2.3. Data Classification

See Table 1 for data classification. Data were extracted from resources using the Population, Intervention, Control, Outcome (PICO) model for clinical evaluation [27]. The target population was identified according to the ONS categories of vulnerability [16]. These are: ‘Psychological Vulnerability’ including acute mental health needs, neurodevelopmental or intellectual disability, or eating, anxiety or mood disorders (Population Area A); ‘Physical Vulnerability’ involving underlying health conditions, chronic pain, respiratory problems, etc., extending to dementia, which is classified here as a physiological condition (Population Area B); and ‘Socioeconomic Vulnerability’ such as situational poverty, deprivation, rural isolation, low income or protected characteristics such as ethnicity, which are associated with inequitable outcomes (Population Area C) [9]. Additional categories of data were used to augment the PICO classifications to draw out particular aspects of the data, such as geographic location, types of research or evaluation methods employed, duration and length of intervention. All data can be found in the Supplementary Materials (Supplementary Table S1).

A considerable amount of contextual literature that did not report interventions or activities was identified within the search. This was incorporated into a narrative review of the impact of the pandemic and the range of responses focused on mental and physical wellbeing. A summary of peer-reviewed and grey, public domain literature (e.g., websites, public information or social media resources) assessed how community activities addressed needs within the three vulnerability categories.

## 3. Results

This review included a total of 256 resources. Fifty-one of these (20 per cent) were peer-reviewed studies, 72 (28 per cent) were grey literature, and 133 (52 per cent) were resources found in the public domain. Peer-reviewed contextual data included literature on the impact of the pandemic on various vulnerable populations—for example, increases in anxiety symptomology as government lockdowns progressed, coping mechanisms utilised by elderly or isolated individuals or barriers to participation in outdoor activities for people with chronic health conditions. Intervention studies and reviews of interventions outlined the efficacy of community activity interventions during the COVID-19 period. Grey literature also provided contextual information such as government and think tank reports on economic or policy impact, and additionally provided information from charities and community organisations on community activities. Public domain resources included a range of community activities providing an overview of the breadth of projects, schemes and community engagement occurring during the pandemic. Hereon, public domain resources identified by this review are marked with ‘R’ and correspond to Supplementary Table S1. Peer-reviewed and grey literature resources are referenced with the same number in both in the Supplementary Table S1 and within the reference list.

### 3.1. Contextual Literature

Sixteen per cent of resources identified were classified as ‘contextual’, i.e., peer-reviewed data that provided an overview of the psychological, physical and social impact of the COVID-19 pandemic. Within Population Area A (Psychological Vulnerabilities), contextual peer-reviewed data reported population trends and regression data on coping mechanisms, poor mental health or increased symptomology in established psychiatric disorders. This body of data revealed growing concerns around the psychological impact of the pandemic in the UK, a country in which increasing case rates of mental health conditions have been noted over the past several years [28]. There additionally appeared to be an abundance of data reporting the increased need for mental health services during the pandemic [28,29,30,31,32,33,34,35,36,37,38,39]. Pierce et al. [28], for example, reported that lockdown-related trigger mechanisms such as entrapment and loneliness were associated with higher rates of depression, self-harm, suicide and overall poorer mental health during the pandemic. These rates may have been due to lockdown lifestyle adaptations such as increased screen time and decreased exercise impacting sleep, increased stress and decreased wellbeing and physical health [40,41,42,43]. In comparison to previous years’ trends, the Office for National Statistics General Health Questionnaire data showed an 8.1 per cent decrease in mental wellbeing between March and June 2020 [44]. The Office for National Statistics longitudinal data showed a significant decrease in mental wellbeing and affect alongside an increase in distress patterns, particularly amongst those in lower socioeconomic brackets, young people and mothers of preschool children, for whom the pandemic was associated with a heavier socioeconomic impact [16]. Several non-governmental organisations additionally reported psychological effects of lockdowns, as well as coping strategies. The Wellcome Trust, for example, captured how arts and creativity, community relationships, philosophy, nature, green spaces, gaming, volunteering, activism and learning were utilised as coping mechanisms for increased anxiety [45,46]. The Royal Horticultural Society, National Trust, Royal Society for the Protection of Birds, 56 Degree Insight and Thrive reported increases in membership interest, alongside increased interest in nature and outdoor activities during the pandemic [47,48,49,50,51]. The Crafts Council noted in its annual report the meditative benefits and increase in craft making during the pandemic [52]. Meanwhile, those with established psychiatric disorders, learning disabilities, neurodevelopmental conditions such as Autism Spectrum Disorder or ongoing treatment for conditions such as eating disorders experienced a severe reduction in access to points of statutory contact [53,54,55]. Services designed to implement salutogenic approaches in the community were reported to be overstretched during the pandemic, having been fragile and at capacity beforehand [56,57]. Such service gaps may be addressed through a participatory approach [58] or digital interventions [59,60].

Categorised within Population Area B (Physiological Vulnerabilities), a considerable amount of contextual literature was found regarding the importance of regular physical exercise in combatting COVID-19, as well as the effect of comorbidity on case rates. Physical activity was reported to be useful in combating diseases associated with increased inflammation, including metabolic and infectious diseases and acute respiratory infections, whilst sedentary lifestyles impacted negatively on general health status and mental health outcomes [61,62,63,64]. COVID-19 is a multi-organ disease in which physical activity and inflammation status is a mediator of symptom severity and cross-organ communication. Adverse viral effects are regulated by skeletal muscle contraction, immune system responses and effects on adipose tissue [63]. Higher body mass index (BMI) is associated with higher disease impact, alongside lower mental health, lower physical activity levels and higher overeating during the pandemic [65], whilst a reduction in inflammation status allowed effective counteracting of COVID-19 infection [63]. Thus, public health messages around staying active were crucial during the pandemic. 

### 3.2. Community Activities within Population Area A: Psychological Vulnerability

A total of 78 identified resources, comprising peer-reviewed and grey literature and public domain resources, addressed psychological vulnerability (Table 2). Resources within Population Area A comprised interventions and activities for individuals with mental health needs, as well as activities and interventions targeting all groups, with general mental health or wellbeing as an intended outcome. The main groups identified or targeted were individuals experiencing addiction, anxiety, cognitive impairment, depression, eating disorders, adults with learning difficulties or Autism Spectrum Disorder.

In response to the issues highlighted by the pandemic, numerous community organisations adapted services to reach out to people with psychological vulnerabilities. At the beginning of the pandemic, community organisations and charities adapted their websites to contain online resources to navigate users towards shopping, test and trace and health services (R68, R69, R72, R73, R75). Others offered information on what to do in the event of a mental health crisis during lockdown (R3) or, in the case of adults with learning disabilities, user-friendly and accessible factsheets outlining the meaning of COVID-19 ‘lockdown’, the need to wash hands and socially distance (R64, R65). The majority (66%) of community organisations moved their offers online. Several used platforms such as Zoom to deliver arts and craft or painting tutorials (R10, R48, R160), photography and mindfulness courses (R146), interpretative dance (R10) or choir practice [66] with the intent of alleviating psychological distress arising from addiction (R24, R48, R73), head injury (R18), palliative care (R18), caregiver burden (R48, R67, R72), postnatal depression (R52) or general wellbeing (R56, R57, R59, R72) and mental health (R1, R10, R18, R29, R48, R49) as intended outcomes. Some community organisations sent ‘creative care packages’ through the post, such as papercraft activity packs containing pencils, paint, coloured paper, glue, stickers and activity work books (R121, R132, R160, R159), as well as music and singing-at-home activity packs (R132). Three community organisations, Beaney House of Art and Knowledge, Look Again South West and Suffolk Art Link, hosted online art tutorials, sent out art packs and presented their users with the opportunity to exhibit artwork in online galleries (R4, R18, R57). One private-sector organisation uploaded pictures and videos of serene railway journeys from around the world in order to promote mindfulness and calm (R127).

Of the 31 community activities identified targeting people with mental health difficulties, three evaluated their activities using validated outcome measurements (R18, R29, R70). Tees, Esk and Wear Valley NHS Trust reported a music and wellbeing programme for NHS staff using validated wellbeing questionnaires alongside regression analysis to measure impact (R29), whilst others, such as Look Again and Performance Medicine, partnered with universities to measure impact (R18, R70). The other 28 community activities either reported outcomes, participant feedback, challenges and successes, survey results or output, including numbers of participants, phone and video calls as measures of impact, but did not employ validated outcome measures. Six reported more generic outcomes, with two stating that their outcomes ‘promote wellbeing’ and ‘support mental health’ (R48, R49) and others suggesting that their offer aimed to promote ‘practical ways to stay connected’ (R69), ‘combat loneliness’ (R72), support the ‘Six Ways to Wellbeing’ framework (R4) or ‘reduce anxiety and increase resilience’ (R34). Four used feedback quotes from participants (e.g., ‘I found the process of drawing and painting both cathartic and healing at the most difficult time of my life’; R30, R52, R56, R59); four reported challenges and successes (e.g., ‘unable to engage with digital content’, ‘offline activity is more labour intensive’; R17, R29, R48, R49); and one community group used their own survey to measure impact (R1). Others reported their outputs as a measure of impact (e.g., ‘we created a new website’; R48) whilst others counted participant numbers and retention (R18, R52, R56) or increased use of phone calls and Zoom meetings (R57).

Peer-reviewed data identified by the review consistently reported increased wellbeing in relation to community activities. Pierce et al. [28] reported that individuals with higher levels of social support were more likely to participate in community volunteering whilst those with diagnosed mental health conditions were more likely to engage in social action volunteering, in contrast to volunteering trends during non-emergency periods [29]. ‘Happiness’ and ‘gratitude’ were significantly associated with nature walks and hiking [67], whilst one meta-analysis [54] reported that self-guided interventions such as Cognitive Behavioural Therapy, mindfulness and acceptance therapy, used alongside music and physical exercise, helped with stress and coping behaviour [54]. Meanwhile, cooking, decorating, diary writing and researching were related to positive emotions [67], whilst amount of gaming time, contrary to popular belief, was slightly but statistically significantly correlated (β = 0.31; R^2^ = 0.15) with wellbeing [68]. Volunteering [56], showing kindness [69], gaming [70], foraging [71], being in nature [72,73], listening to music [74], exercising [75], sewing [76] and engaging in arts and crafts [77], was shown to positively impact wellbeing during the pandemic.

Further, peer-reviewed evidence suggested that interaction with nature increased during lockdown, with 60–72 per cent of one large-scale survey of 703 UK adults reporting an increased desire to spend time amongst nature, with 94 per cent of this sample recording that they had heard more birdsong, with benefits of noticing nature described as: ‘mindful’, ‘liberated’, ‘togetherness’ or ‘self-worth’ ([51], p. 9). Table 3 outlines the major and minor themes organised by PICO for Population Area A.

### 3.3. Community Activities within Population Area B: Physical Vulnerability

Seventeen percent of all resources identified were categorised into Population Area B: Physical Vulnerability. The largest number of these were aimed towards individuals with dementia; these are reported separately below. The remaining resources were targeted towards participants with physical health conditions and focused on individuals who were shielding, with immunocompromising conditions or living with chronic pain. Other resources were aimed at a wider audience but focused on different physical interventions such as singing for lung health, exercise, activity or dance.

The pandemic increased public park visits and highlighted the need for more green spaces to be integrated into the urban infrastructure [78,79]. Operational changes and upheaval to exercise referral schemes impacted mental health, particularly due to pandemic-related restrictions and a lack of available exercise [61,80]. According to a cross-cultural comparison study [81], lower levels of exercise were associated with poorer mental health outcomes in the UK, Ireland, New Zealand and Australia, with the younger age category of 18–29-year-olds showing the largest decrease in physical activity out of any of the measured demographics [61]. Similarly, a longitudinal study with almost 6000 participants [82] found a population-wide 63 per cent decrease in physical activity during the pandemic, with high-income earners increasing activity levels, and younger age groups showing the highest reduction in physical activity [82]. Sport England reported ‘unprecedented’ drops in physical activity amongst its survey of 2000 UK adults [83], which coincides with increased levels of overeating behaviours [65]. Conversely, younger people were most likely to engage in more intense physical exercise, with confounding factors being access to outdoor space, higher income and being female. Those with obesity, hypertension, lung disease and living alone appeared less likely to change their physical activity habits [80].

The literature reported several physical exercise, outdoor activity, dance and movement-related activities that were established during the pandemic that were aimed both at individuals who were shielding due to underlying health conditions, and the community as a whole. Online yoga classes, for instance, had positive effects on pain intensity, anxiety and depression [84], whilst interpretative dance practice generated feelings of collectiveness and cultural togetherness [85], although the challenges of teaching and limited proximity raised concerns around the equity of access. There were mixed reports on the impact of arts-based activities on physical health. For instance, singing during the pandemic helped improve lung health, depression and confidence but not other psychological or health measures, including physical function, energy, emotional wellbeing, pain, social function, general health or health change over the past year [86]. In-person singing additionally was found to increase the aerosol risk of transmitting COVID-19 [87].

Within the community, grey literature and public domain resources described various arts-based interventions. Escape Arts (R12) and University of Cambridge Museums (R58) sent out creative art packs, physical resources and family activity ideas to parents who were shielding and parents of children with terminal illness. Several community organisations organised live music, including classical concerts and choirs (R116, R172), online exhibitions for shielding individuals to display their work (R4, 13), art on windows (R19), at-home museum collections and crafts (R23), food creativity and world culture (R27) and drama, entertainment and doorstep theatre (R28) for individuals who were shielding.

#### Dementia

Fifteen of the 39 items identified by the review pertaining to individuals with physical health conditions were targeted at individuals with dementia and their caregivers, owing presumably to the impact of the pandemic on this population. A national survey reported that the public health restrictions reduced day-to-day access to statutory social support services, and social activities in the community such as choirs, reading groups and befriending services, and were negatively associated with the mental health and wellbeing of older people, people with dementia and their caregivers [88].

Alzheimer’s UK published online resources for people with dementia and their caregivers, outlining available support within hospitals and care homes as well as general information on Coronavirus and its effect on individuals with dementia (R71). Similarly, the Alzheimer’s Society published positive mental health resources for individuals with dementia and their carers, as well as advice on shopping, leaving home and safeguarding; music and reminiscence activities were published on their website with large fonts and an accessible user interface (R144). Reminiscence resources were also published by the BBC and the Museum of London in 2020, providing visual prompts for individuals with dementia to remember and reflect on the past by scrolling through archival film footage of the twentieth century (R21, R149).

There is evidence to suggest that wellbeing can be enhanced through community-based arts activities, which can create feelings of social connection, happiness and rejuvenation [89]. Community organisations targeted at individuals with dementia sent out visual art, arts and crafts creativity packs and performed regular telephone check-ins (R21, R22, R38, R45, R47), whilst others such as Acto Dementia used in-community focus groups to test and recommend art, gardening, sports or boardgame touchscreen apps to aid with activity setting during self-isolation (R145). Museum and social prescribing resources were also made available either through online weekly meetups or signposting to remote access art events aimed for people with dementia (R147, R51). Three community organisations identified offered online weekly workshops: the Garden Museum offered ‘Clay for Dementia’, an eight-week pottery class (R50); Aspex offered a weekly art workshop over Zoom (R43); and Lost in Art (R47) has been delivering visual arts-based activities during the pandemic. Table 4 outlines the major and minor themes organised by PICO for Population Area B.

### 3.4. Community Activities within Population Area C: Socioeconomic Vulnerabilities

This review sought to assess the extent to which health inequity was addressed by community initiatives. The majority of the literature found was based on community initiatives or interventions around social isolation, loneliness and community togetherness, but only 16.5% (17 out of 103) of articles were aimed specifically at individuals in deprivation categories. Higher rates of COVID-19-related impact amongst individuals in the more deprived categories highlight social and regional health inequity and a social gradient in health outcomes [7], whilst policy and societal responses will largely determine future health, wellbeing and economic outcomes for individuals in these deprived and protected categories [9]. Arts on prescription and leisure initiatives can address health inequity [90,91] but there is a long way to go, particularly given that engagement in arts activities (as well as the availability of such resources) is limited and influenced by social and geographic factors [31].

The most obvious starting point for this hand search was found within the resources aimed at ameliorating financial worries for low-paid workers, small business owners and third-sector organisations. Financial and business advice for arts professionals was found (R86) alongside many microsites outlining aid to recovery, help with universal credit, ‘staying well, supported and creative’ during the pandemic, as well as resources for networking, online collaboration, contingency planning or mental health advice for students and young renting professionals on low incomes (R77, R79, R80, R81-R83, R87, R88). The National Council for Voluntary Organisations offered business advice, identifying risk and resources on managing budgeting and staffing (R85), and the National Endowment for Science, Technology and the Arts published a repository of advice and funding avenues for small businesses (R84).

The majority of arts-based community resources identified (58 out of 103) were targeted towards isolated and lonely individuals. Creative and arts-based interventions included initiatives such as communal art, community notice boards, chalk murals, online arts and crafts clubs, participatory arts projects (R89–R93, R96–R98, R100–R104, R110, R111, R151), online workshops, digital art creative community for carers, at-home DIY art kits, block printing, art tutorials, stand-up comedy, reading groups, heritage from home and art from home resources (R3, R25, R39, R105, R110, R117, R118, R119, R120, R121, R125, R128, R164). Others provided singing workshops, neighbourhood singalongs and sing for better breathing workshops (R95, R112, R139, R140). A number of music-related provisions were also offered for isolated individuals, such as back garden gigs, online jamming sessions, dance live streams and a BBC orchestra for isolated people (R60, R99, R124, R130, R132). Nature activities for isolated individuals focussed on foraging, outdoor hiking ideas and wildlife webcams (R36, R42, R94, R135, R142, R143).

Seventeen resources were identified that were targeted towards individuals experiencing deprivation, themed into: abuse, asset poor, care leavers, families in chronic crisis, digital poverty. For those experiencing abuse, Creative Learning Guild sent out arts-based creative care packages, which were praised by social workers as promoting family togetherness and reducing stress (R9). Two organisations, the Wildlife Trust and Outdoors for All, set up webcams in natural environments to enable people in urban areas to access green spaces and nature digitally; however, evaluation and feedback was not available for either activity (R36, R42). Collective Encounters published a report on the positive role of participatory theatre on social change, but challenges remain in reaching vulnerable audiences, such as those experiencing situational and digital poverty, for whom online activities are difficult to engage in [92]. During the pandemic, a number of novel partnerships within the community arose between cultural organisations and local risk registers. ‘No-one in Holbeck and Beeston Goes Hungry’, for example, was a community scheme in Leeds in which a food bank and theatre were established within a working men’s club (R179), whilst another organisation, the Old Courts Arts Centre in Wigan, used their event and management logistics, existing technology and furloughed workers to turn the arts centre into a food bank warehouse and distribution centre for the community (R182). Another local authority organisation, FEAST, utilised out-of-work artists to work within deprived communities in Cornwall (R181). Create is a national organisation that reached out to young carers and their families during the pandemic through photography, dance, drama and music workshops run by artists over four weeks of lockdown (R8), and Coram—Letters in Lockdown provided writing workshops for young carers and their families (R7). Both Create and Coram used novel (unnamed) evaluation questionnaires and feedback quotes to evaluate their services. Everyone Connected and the Arts Council offered support for communities experiencing digital poverty through access to accurate health information online, allowing interaction with medical support, using essential services and allowing individuals to stay locally connected (R61, R62, R157) and the Arts Council through developing digital skills, networking and digital training. Resources for prison staff and voluntary organisations working with people in the criminal justice system both within prison and in the community were available (R74). Table 5 outlines the major and minor themes organised by PICO for Population Area C.

### 3.5. Quality Assessment of Literature

Due to the varied nature of the reviewed literature, several quality assessment frameworks were used to evaluate quality. Examples of activities, case studies that formed part of grey literature resources and public domain resources were quality-assessed using the Arts for Health and Wellbeing evaluation framework [23]. Broadly speaking, the assessment fell into two categories—essential information (for example, aims and objectives, commissioner and funding sources, timescales, exposure, type of intervention, quality assurance) and evaluation details (evaluation aims, type of evaluation, evaluation design). Put together, this information can paint a picture of the impact and need for the intervention. Peer-reviewed data were quality-assessed using Cochrane and AMSTAR evaluation methods, whilst grey literature was measured using the AACODS critical appraisal tool. In conducting quality assessments, it may be possible to evaluate whether efficacy claims could be validated with a robust methodological framework. As Table 6 shows, whilst a number of peer-reviewed and grey literature articles were identified, less than half of the identified literature met quality thresholds.

## 4. Discussion

### 4.1. Summary of Findings

This review identified 256 resources comprising peer-reviewed articles, grey literature and information within the public domain. Excluding contextual data, the review found 217 peer-reviewed, grey literature and public domain items. Peer-reviewed data included interventions, randomised controlled trials and reviews of interventions focusing on community engagement supporting health outcomes. Grey literature resources included non-peer-reviewed reports and case studies conducted by charities, governmental departments and university research departments. Resources in the public domain provided examples of community engagement such as mutual aid groups and online community activities. The review identified a large number of arts, creative, crafts, textiles and embroidery, gardening and nature, music, theatre, exercise, board-gaming and social activities designed with the intention of ameliorating negative social health outcomes. The majority of resources were aimed either at wellbeing and positive mental health, or social isolation and loneliness. Whilst it is clear that an abundance of activity materialised during the pandemic, its impact and efficacy is less clear. Most peer-reviewed literature fell within the Cochrane ‘Bronze’ category, with 11 per cent attaining ‘Silver’ and none attaining ‘Gold’. Of the three articles assessed using AMSTAR, two were ‘Critically Low’ and one was ‘Moderate’. Only a third of the grey literature and public domain resources met the PHE threshold for quality, whilst half met the quality criteria for grey literature. Within the case study, grey and public domain literature, articles were more likely to be of better quality if they had completed or published evaluation methods—even if those methods were not established or validated.

### 4.2. Discussion of Findings

The aim of this review was to understand the scope, efficacy and nature of community-based interventions for vulnerable and non-vulnerable populations during the pandemic. A large number of community initiatives were found seeking to mitigate social, physical and psychological vulnerabilities during the pandemic. A minority provided effective reach to vulnerable populations using established evaluation methods to validate their data; however, there is need for more high-quality literature on the role of community activities in the mitigation of vulnerabilities.

Salutogenic approaches are strongly supported by the evidence base, particularly in regard to their positive psychological, psychological, social and behavioural outcomes [1,4,5]. Whilst a large number of community initiatives were identified within the present search, there was a lack of clarity regarding the impact of initiatives, with many providing anecdotal evidence only, and with few employing validated wellbeing measures. Consequently, this review found a lack of validated efficacy and impact data regarding the benefits of salutogenic approaches on vulnerable individuals during the pandemic. Within each resource identified, the authors firstly assessed whether evaluation methods were reported on at all, and secondly assessed the validation of the evaluation methods. In some instances, it was not always clear whether an organisation completed an internal evaluation, or perhaps whether there was need for one when considering micro-organisations such as mutual aid groups. Future research in this area should consider the value of different types of evaluation methods, particularly those that are straightforward for organisations to report. The PHE Arts for Health and Wellbeing Evaluation Framework [23], for example, can be used to validate qualitative feedback such as reporting on the challenges and successes of projects, and participant feedback, or reporting whether quality assurance standards were met (for example, within the design and professional delivery of the activity). Such measures, when used by community organisations, can contribute valuable evaluation data and contribute to the measurement of impact and efficacy. Furthermore, evidence of intervention success was also lacking within the identified literature. Only eleven of the peer-reviewed resources were intervention studies assessing the health impact of community engagement, all of which reported on anxiety, coping, wellbeing, dementia symptomology and chronic pain. None assessed socioeconomic vulnerabilities.

Overwhelmingly, the peer-reviewed literature reported the positive impact of community engagement on mental health and wellbeing; however, several limitations were found, primarily with generalisability to the overall population and internal bias relating to the reliability of data. In some instances, the sample participants were weighted in age, socioeconomic status and digital literacy and not representative of the general population. One study conducted by Loynes [78], for example, reported that 94% of participants had heard more birdsong during the first lockdown. A potential sample bias in this study was that the online, older age and higher socioeconomic status-weighted sample was not representative of the general population. Those living in densely populated areas, in high-rise buildings or with less access to outdoor space may have differing experiences of (or access to) nature, which was not reported in the study. This is particularly important when assessing items that are dependent on demographic data such as community engagement. An article by Johannes, Vuorre and Przybylski [68], as another example, reported that higher levels of mental wellbeing were associated with longer video gameplay; however, the research, which was commissioned by EA Games and Nintendo, only focussed on two ‘light touch’ games aimed at children, which were analysed within their sample of adults and so were not representative of gaming, which is normally associated with negative outcomes [68].

From the content of the data that were collected, it may be the case that salutogenic approaches occur alongside and reinforce traditional community and public health approaches rather than replace them. For instance, charities working with adults with learning difficulties (R64–R68, R72) extensively disseminated public health information regarding the new virus, washing hands, social distancing, how to get help if an individual feels unwell, etc., alongside coping strategies, meditation and other such holistic advice. The dissemination of public health information regarding the new virus was essential information for this community, as were holistic interventions targeting mindfulness and coping mechanisms.

The impact of community engagement on socioeconomically vulnerable populations remains less clear, mitigating pandemic impacts. It is already known that lower levels of wellbeing and socioeconomic positions are individually associated with lower levels of cultural engagement [31]. Fancourt and Baxter [31] explain this discrepancy within the Capabilities, Opportunities and Motivations framework [93]: physical opportunity, educational attainment and area deprivation are contributory factors in that they provide barriers to participation. Individuals may face financial barriers, digital poverty, have more pressing work or caring commitments, be in rural areas with less access to transport or not have the inclination or trust in participating [31]. Meanwhile, the risks of mortality for COVID-19 are cumulative. They include: being male, older, an ethnic minority, having an underlying health condition, working in a higher-risk occupation and living in deprived areas [7]. Those experiencing the highest levels of inequity have been more adversely affected by COVID-19 [7], yet only ten per cent of this review’s total identified resources explicitly targeted individuals experiencing deprivation, digital poverty or low income, with the majority of socioeconomic outcomes assessing social isolation and loneliness. A number of resources were identified offering advice on housing, universal credit and other statutory services associated with lower incomes, but the review identified only fourteen community activities for people in deprivation categories, the majority of which were online art and nature resources and craft workshops, with three currently working on validated measurements (as yet unpublished), and four using participant feedback, unnamed evaluation questionnaires or reporting on strengths and challenges. It was therefore unclear whether community activities were efficacious within areas of deprivation. In addition, the target populations often overlapped between the three population areas, which demonstrates some of the associational effects of socioeconomic status on health and psychology. Given that vulnerable populations living in areas of deprivation appear to be more adversely affected by health inequity [9] and that community engagement can promote healthy psychological, physiological, social and behavioural responses [31,94], there is immediate value therefore in using community assets to engage with vulnerable groups. Community engagement can offer multiple avenues to support complex needs and has the potential to be particularly useful in promoting wellbeing within communities.

### 4.3. Limitations

Several limitations to this review should be considered. Firstly, it is systematised but not systematic due to the diversity of resources, which span peer-reviewed, non-peer-reviewed and public domain information. It was beyond the scope of this review to capture all community activities during the COVID-19 pandemic. It therefore offers a broad sample of the available evidence. Within the included studies, several instances of reporter, geographical or socioeconomic bias were identified. Whilst this is a wider limitation of the field of study, it must be noted here that more information on the accuracy and generalisability of studies should be taken into consideration. Unlike other reviews, the present study includes both interventions and non-interventions, whilst the contextual information provides a picture of the impact of the pandemic as well as the emergent and urgent concerns that ought to be addressed by community interventions.

## 5. Conclusions

The aim of this review was to draw together a timely summary of how community activities have been utilised between March 2020 and January 2021. The review examined how formal and informal community organisations, charities, community assets and mutual aid groups formed ad hoc services to support individuals. Some were formed with the specific aim of supporting vulnerable individuals through the pandemic, whilst others were aimed at supporting the social, physical and mental wellbeing of the community as a whole.

It is important within this field of study to understand the efficacy of such services, including short-term and long-term impacts. Existing research has shown that participation in arts, nature, music, cultural and physical activities is key to public health and wellbeing. This review found an abundance of resources and reports of cultural engagement amongst UK communities in response to the COVID-19 pandemic, with diverse foci and design principles. Given the timescale, it is unlikely that there will be robust long-term evidence of the efficacy of these initiatives. There is a need therefore for further research, particularly on the efficacy of arts participation in addressing inequalities and supporting vulnerable populations.

## Figures and Tables

**Table 1:** Data classification, type and PICO.

**Data Classification**		
Peer-reviewed literature (contextual literature, intervention studies; review of interventions) Grey literature (evaluation reports; NGO reports; government reports; charity reports) Public domain (charity webpage; mutual aid resources; community outreach; commercial; social media posts)		
**Type of Resource**		
E.g., charity project, CIC project, cultural asset case study, example of activity, online resource, project case study, RCT, cross-sectional study, longitudinal study, population analysis, service evaluation, thought piece, unpublished study.		
**Population**	*Vulnerability/RER theme*	Psychological/Socioeconomic/Physiological/Other.
	*Subtheme*	E.g., Addiction, caregiving, chronic pain, coping, deprivation, digital poverty, eating disorder, elderly, everyone in the community, low income, resilience, shielding.
	*Target Population*	E.g., All/everyone in the community, adults with mental health issues, careleavers, dance professionals, disabled artists, hospital patients, people using foodbanks, prison staff, people experiencing chronic pain, members of local choirs.
**Intervention**	*Type of intervention*	E.g., Animals, creative, cooking, cultural, gaming, gardening, music, poetry, protest, movement, dance, sewing/embroidery, museums.
	*Method of delivery*	E.g., online, posted, door to door, over Zoom.
	*Exposure*	E.g., One hour
	*Frequency*	E.g., Once a week
	*Duration*	E.g., Over the period of three months; over the duration of lockdown
	*Quality assurance*	E.g., Therapist-led, mental health professional-led, professional musician-led, professional artist-led.
	*Cost to participant*	E.g., No cost to participant, membership fee of GBP 5 per month
	*Geographical location*	UK-wide, Northern Ireland, Scotland, NE England, NW England, Central England, Wales, Midlands, South East England, South West England, Greater London, International (but applicable to UK population)
**Control**	*Was a control group used?*	No control group/Control group (n)/Comparison data
**Outcome**	*Aims/objectives*	Eg., Reach out to BAME audience, combatting isolation, improve health and wellbeing, improve access to service.
	*Evaluation methodology*	E.g., anecdotal, feedback forms, evaluation questionnaires, validated measurements (e.g., WEMWBS), formal evaluation in partnership with a university, statistical evaluation, no evaluation used.
	*Outputs*	E.g., events, exhibitions, online gallery, website, number of sessions. Some comlpeted, some intended.

**Table 2:** Number of resources within each type of literature.

	Population Area A—Psychological (% Total)	Population Area B—Physiological (% Total)	Population Area C—Social (% Total)	Other Populations (% Total)	Open to All (% Total)	Total (% of Total)
Peer-reviewed (contextual literature)	23	11	1	1	4	40 (16%)
Peer-reviewed (intervention study)	4	4	0	0	0	8 (0.03%)
Peer-reviewed (reviews)	3	0	0	0	0	3 (0.01%)
Grey literature	26	19	23	0	4	72 (28%)
Public domain	22	9	71	6	25	133 (52%)
Total	78 (30.4%)	43 (16.8%)	95 (37.1%)	7 (0.03%)	33 (12.9%)	256

**Table 3:** Major and minor themes organised by PICO for Population Area A.

	Population (% Resources)	Intervention (% Resources)	Control (% Resources)	Outcome (% Resources)
**Major theme**	All individuals with general mental health or wellbeing as an outcome (56%)	Art/Creativity (27%)	No Controls (100%)	Wellbeing (28%)
**Minor themes**	Learning difficulties (11%), Eating disorders (5%), Anxiety (4%), Other (24%).	Nature (13%), Gaming (3%), Gardening (3%), Other (54%).		General mental health (25%), Other (47%)

**Table 4:** Major and minor themes organised by PICO for Population Area B.

	Population	Intervention	Control	Outcome
	(% Resources)	(% Resources)	(% Resources)	(% Resources)
**Major Theme**	Dementia (35%)	Creative/Art (51%)	No control (91%)	Improve health and wellbeing (58%)
**Minor Themes**	Shielding (16%), Physical activity (12%), Other (37%)	Music (14%), Exercise (14%), Other (21%)	Control RCT (*n* = 4; 9%)	Reduce isolation and loneliness (32%), Other (10%)

**Table 5:** Major and minor themes organised by PICO for Population Area C.

	Population	Intervention	Control	Outcome
	(% Resources)	(% Resources)	(% Resources)	(% Resources)
**Major Theme**	Isolated (53%)	Creative/Art (73%)	No control	Improve community cohesion (56%)
**Minor Themes**	Deprivation (15%), Low income (10%), Older (6%), Other (16%)	Music (12%), Nature (4%), Other (11%)	-	Isolation (8%), Other (36%)

**Table 6:** Quality appraisal of resources.

Appraisal Tool	*Resources (N)*
**Cochrane (total applicable)**	**50**
Cochrane Bronze	45
Cochrane Silver	5
Cochrane Gold	0
**AMSTAR (total applicable)**	**3**
AMSTAR High	0
AMSTAR Moderate	1
AMSTAR Low	0
AMSTAR Critically Low	2
**PHE (total applicable)**	**181**
PHE > 10	54
**AACODS (total applicable)**	**22**
AACODS > 22/34	11

## Data Availability

Not applicable.

## Supplementary Materials

The following supporting information can be downloaded at: https://www.mdpi.com/article/10.3390/ijerph19074086/s1. Table S1. The data of PICO (Population, Intervention, Control, Outcome).

## Appendix A

### Appendix A.1. Inclusion Criteria

Types of studies: creative, arts and nature-related activities and resources created or changed in response to the protecting individual wellbeing and health during the COVID-19 pandemic.

Time Period: March 2020–January 2021

Language: English

Demographic Target: UK Adults

Research type: (In order of priority)

Peer-reviewed and academic journals, governmental reports, organisational reports, grey literature including online case study examples.

Primary Databases:

Google Scholar, PubMed, Web of Science, Taylor and Francis, The Lancet, MedRXic, PsycharXiv.

### Appendix A.2. Exclusion Criteria

Studies on creative activities unrelated to COVID-19 pandemic and/or wellbeing

Studies not generalisable to UK Adults, or focused only on vulnerable groups with specific needs, as these require specific interventions that are not generalised to the wider population.
